# Primary acquired spondylodiscitis shows a more severe course than spondylodiscitis following spine surgery: a single-center retrospective study of 159 cases

**DOI:** 10.1007/s10143-017-0829-9

**Published:** 2017-02-27

**Authors:** Anja Tschugg, Sara Lener, Sebastian Hartmann, Andreas Rietzler, Sabrina Neururer, Claudius Thomé

**Affiliations:** 1 Department of Neurosurgery, Medical University of Innsbruck, Anichstr. 35, 6020 Innsbruck, Austria; 2 Department of Neuroradiology, Medical University of Innsbruck, Innsbruck, Austria; 3 Department of Medical Statistics, Informatics and Health Economics, Medical University of Innsbruck, Innsbruck, Austria

**Keywords:** Postoperative spondylodiscitis, Primary spondylodiscitis, Discitis, Epidural abscess, Spinal infection

## Abstract

Spondylodiscitis may arise primarily via hematogenous spread or direct inoculation of virulent organisms during spine surgery. To date, no comparative data investigating the differences between primary and postoperative spondylodiscitis is available. Thus, the purpose of this retrospective study was to investigate differences between these two etiologies. One hundred fifty-nine patients that were treated at our department were included in the retrospective analysis. The patients were categorized into two groups based on the etiology of spondylodiscitis: group NS, primary spondylodiscitis without prior spinal surgery; group S, spondylodiscitis following spinal surgery. Evaluation included magnetic resonance imaging (MRI), laboratory values, clinical outcome, and operative or conservative management. Preoperative MRI showed higher rates of epidural and paraspinal abscess in patients with primary spondylodiscitis (*p* < 0.005). Vertebral bone destruction was more severe in group NS (*p* < 0.05). Survival rate in group S (98.2%) was higher than in group NS (87.5%, *p* = 0.024). The extent of the operative procedure in patients who were surgically treated (*n* = 116) differed between the two groups (*p* < 0.005). In conclusion, spondylodiscitis is a life-threatening and serious disease and requires long-term treatment. Primary spondylodiscitis is frequently associated with epidural and paraspinal abscess, vertebral bone destruction and has a higher mortality rate than postoperative spondylodiscitis. Therefore, primary spondylodiscitis shows a more severe course than spondylodiscitis following spine surgery.

## Introduction

The incidence of spondylodiscitis is rising probably due to an aging population, chronic immune-compromising diseases, frequent spine procedures, and the incessant advancement of diagnostics [1]. A spondylodiscitis may arise primarily through hematogenous spread or direct inoculation of virulent organisms during spine surgery, epidural injections, nerve root block, or discography [2]. Patients suffering from spondylodiscitis have increased long-term mortality. Thus, it is a life-threatening and serious disease [3]. Due to the increasing incidence of spondylodiscitis, there is an urgent need for further investigations. To best of our knowledge, no comparative data examining potential differences between primary and postoperative spondylodiscitis is available. Thus, the purpose of the present retrospective study was to detect differences between these two etiologies in clinical features, magnetic resonance imaging (MRI), outcome, and operative management.

## Material and methods

**Fig. 1:**
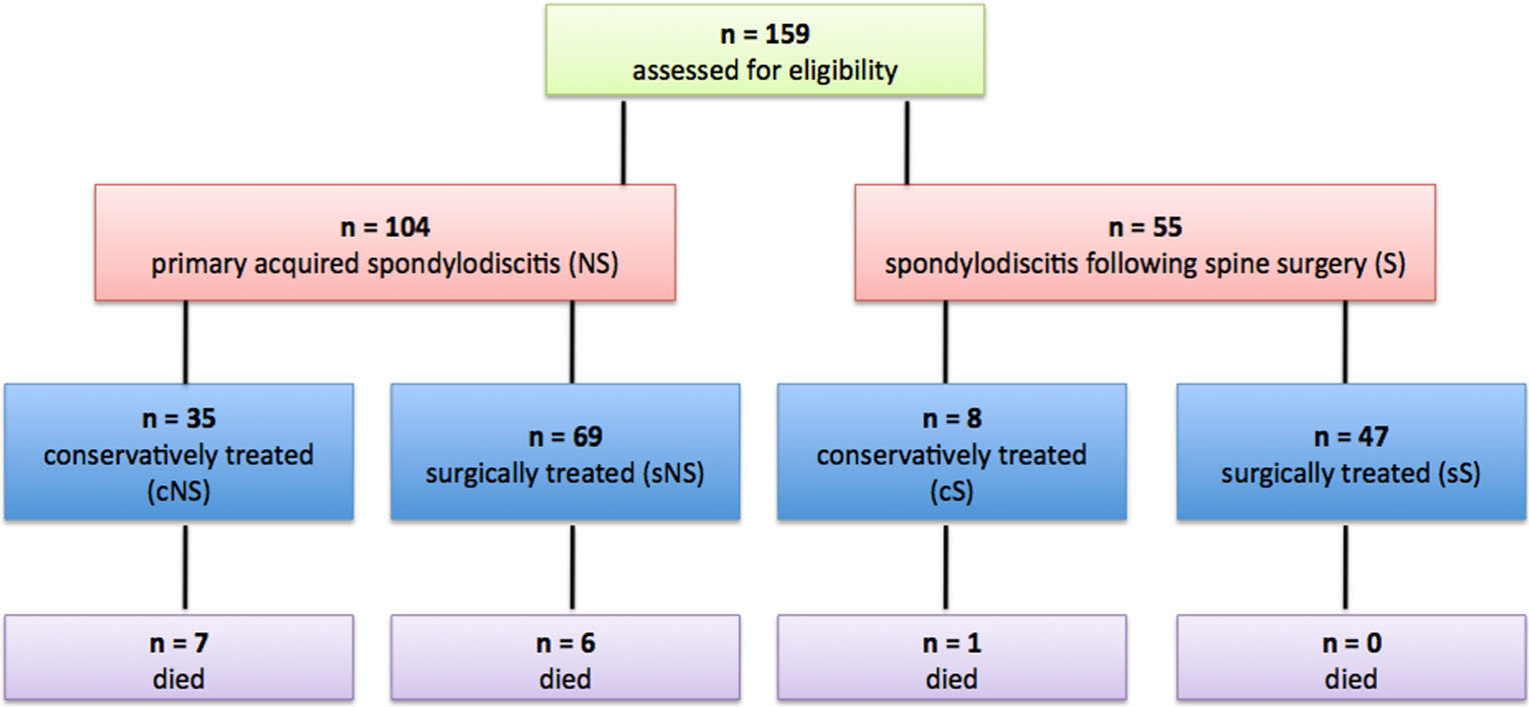
Flow chart on the treatment in all patients with primary (NS) and secondary acquired spondylodiscitis (S). *n* number of patients, *c* conservatively treated, *s* surgically treated

A retrospective review of patients who underwent treatment for spondylodiscitis between 2010 and 2016 at our department was performed. One hundred fifty-nine patients were identified and their data were retrospectively investigated. The patients were then categorized into two groups based on the etiology of spondylodiscitis: group NS, primary spondylodiscitis without prior spinal surgery; group S, spondylodiscitis due to spinal surgery. Data were collected using the patients’ health records and MRI. MRI was read by an independent neuroradiologist, blinded to the patients’ clinical data. C-reactive protein (CRP) and complete white cell count were analyzed routinely. Patients with neurological deficits, progressive pain, or progression in MRI despite conservative treatment underwent surgical treatment. Each treatment option was accompanied by broad-spectrum antibiotic therapy or antibiotics according to the antiobiogram when available. Patients were treated with intravenous broad-spectrum antibiotics for a short period of time (2 to 6 weeks) followed by oral antibiotics for a total period of 3 months. After initial diagnosis, data of conservatively (cS, cNS) or surgically (sS, sNS) treated patients were analyzed separately (Fig. 1). In surgically treated patients (*n* = 117), the two groups were compared at the time of the initial diagnosis, the day of admission, on the third postoperative day, before discharge, and after 6 and 12 months follow-up. The surgical approach and technique was determined for each patient individually according to the present comorbidities, location, extent of infection, and bony destruction. Options used are described in Table 1. In conservatively treated patients (*n* = 43), the two groups were compared at the time of initial diagnosis, 3 and 14 days, and 1.5 and 3 months after initial diagnosis. Long-term follow-up was not available in conservatively treated patients, as follow-up is usually terminated as soon as CRP values normalized and clinical complaints are relieved.

**Table 1:** The surgical approach and method of spinal surgery in patients with primary and secondary acquired spondylodiscitis that were surgically treated

	Group sS *n* = 47	Group sNS *n* = 69
Cervical fusion—anterior, *n* (%)	1/47 (2)	4/69 (6)
Cervical fusion—posterior, *n* (%)	1/47 (2)	2/69 (3)
Cervical corpectomy without dorsal instrumentation, *n* (%)	0/47 (0)	8/69 (11)
Cervical corpectomy with dorsal instrumentation, *n* (%)	1/47 (2)	2/69 (3)
Thoracic fusion, *n* (%)	4/47 (8)	9/69 (13)
Thoracic corpectomy with instrumentation, *n* (%)	1/47 (2)	6/69 (9)
Lumbar instrumentation, *n* (%)	35/47 (74)	32/69 (46)
Lumbar corpectomy with instrumentation, *n* (%)	2/47 (4)	2/69 (3)
Lumbar anterior instrumentation, *n* (%)	0/47 (0)	1/69 (1)
Debridement without instrumentation, *n* (%)	2/47 (4)	3/69 (4)

Corpectomies were more often performed in primary spondylodiscitis

*n* number of patients, *sS* secondary acquired spondylodiscitis surgically treated, *sNS* primary acquired spondylodiscitis surgically treated

## Statistical analysis

All patients with complete initial data were considered for inclusion in the retrospective analysis. All values are expressed as mean ± SD. The Kolmogorov-Smirnov test was used for testing for normal distribution. The unpaired Student’s *t* test and Mann-Whitney *U* test were used to analyze differences in clinical and demographic characteristics and in clinical outcome variables. Frequencies were compared by chi-square and Fisher’s exact tests. Spearman’s rho correlation (*r*) was performed to assess the relation of clinical outcome and MRI findings. A *p* value <0.05 was considered statistically significant. All statistical evaluations were performed with SPSS Version 21.0 (IBM Corp. Released 2012. IBM SPSS Statistics for Windows, Version 21.0, NY: IBM Corp.). Figures were designed using GraphPad Prism (version 5.0 for Mac OS X, GraphPad Software, La Jolla, CA, USA, www.graphpad.com).

## Results

The demographic details and patients’ characteristics are presented in Table 2. One hundred fifty-nine patients who underwent surgical and conservative treatment for spondylodiscitis have been identified at the Department of Neurosurgery. Thereby, the proportion of spondylodiscitis following surgery was 35% (group S, *n* = 55) versus 65% (group NS, *n* = 104) for primary spondylodiscitis. Altogether, 73/159 (46%) patients were female. The most common ASA score was ASA 3° in both groups (*p* > 0.05). Drug abuse was more common in group NS (23/104 (22%); *p* = 0.041). A dorsal decompression was initially performed in 67% of patients in group S. The infection was mostly located in the lumbar spine followed by the thoracic and cervical spine.

**Table 2:** Demographic details in all patients with primary and secondary acquired spondylodiscitis

		Group S *n* = 55	Group NS *n* = 104	Sig.
Age	In years	66.4 ± 11	64.6 ± 12	n.s.
Female gender, (%)		29/55 (52)	57/104 (54)	n.s.
BMI		26.2 ± 3	26.0 ± 6	n.s.
ASA score, *n* (%)	1°	4/55 (7)	10/104 (9)	n.s.
	2°	21/55 (38)	24/104 (23)	n.s.
	3°	28/55 (51)	61/104 (58)	n.s.
	4°	2/55 (3)	9/69 (8)	n.s.
Duration of hospital stay	In days	15.8 ± 10.1	10.5 ± 9.7	0.000
Comorbidities	Depression, *n* (%)	9/55 (16)	6/104 (6)	0.030
	Renal failure, *n* (%)	7/55 (12)	18/104 (17)	n.s.
	Diabetes, *n* (%)	10/55 (18)	19/104 (18)	n.s.
	Heart diseases, *n* (%)	19/55 (34)	35/104 (34)	n.s.
	Hepatopathy, *n* (%)	6/55 (11)	19/104 (18)	n.s.
	Dental disease, *n* (%)	1/55 (2)	6/104 (6)	n.s.
Addiction	Smoking, *n* (%)	11/55 (20)	15/104 (14)	n.s.
	Cigarettes per day	3.9 ± 10	3.0 ± 8	n.s.
	Alcohol, *n* (%)	10/55 (18)	33/104 (32)	n.s.
	Drug abuse, *n* (%)	5/55 (9)	23/104 (22)	0.041
Initial operative procedure	Dorsal decompression, *n* (%)	37/55 (67)	0/104 (0)	
	Dorsal fusion surgery, *n* (%)	13/55 (24)	0/104 (0)	
	Cervical fusion (Cloward), *n* (%)	3/55 (5)	0/104 (0)	
	Kyphoplasty, *n* (%)	2/55 (4)	0/104 (0)	
Location of spondylodiscitis	Cervical, *n* (%)	3/55 (5)	18/104 (17)	n.s.
	Thoracic, *n* (%)	8 /55 (14)	23/104 (22)	
	Lumbar, *n* (%)	42/55 (76)	52/104 (50)	
	Cervical and thoracic, *n* (%)	0/55 (0)	1/104 (1)	
	Cervical and lumbar, *n* (%)	0/55 (0)	1/104 (1)	
	Thoracic and lumbar, *n* (%)	2/55 (4)	9/104 (9)	

*n* number of patients, *S* secondary acquired spondylodiscitis, *NS* primary acquired spondylodiscitis, *Sig*. significant differences, *n*.*s*. not significant

**Fig. 2:**
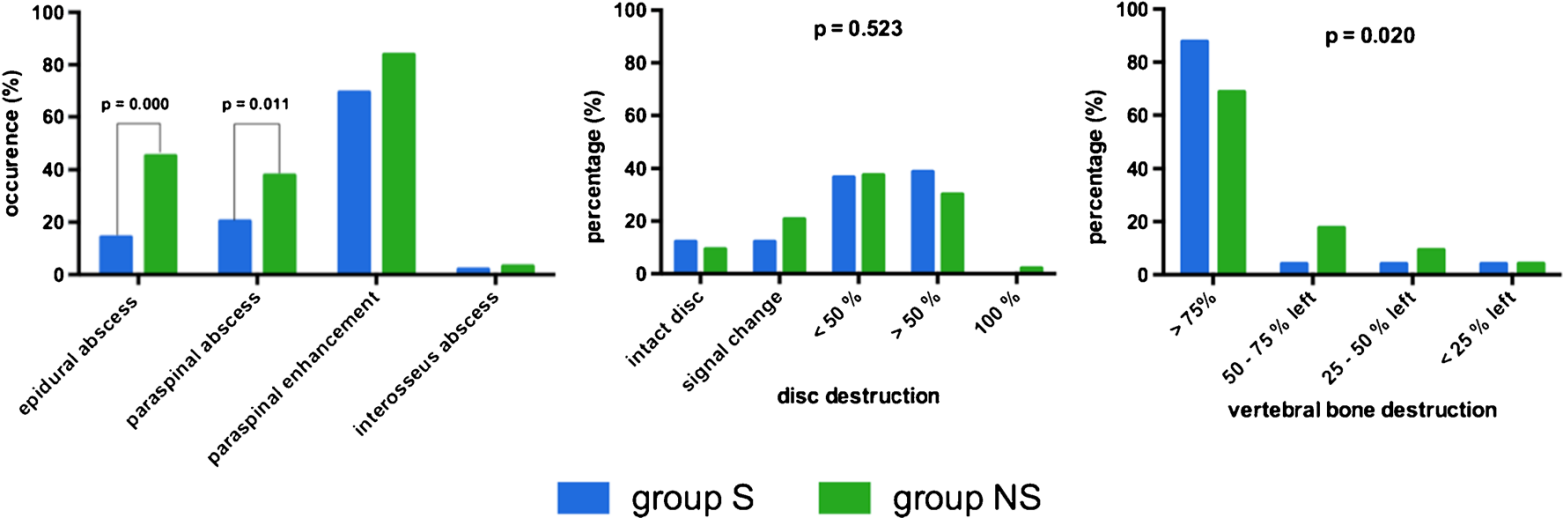
Preoperative magnetic resonance imaging finding in all patients. *S* secondary acquired spondylodiscitis, *NS* primary acquired spondylodiscitis, *p* significance

Preoperative MRI showed higher rates of epidural and paraspinal abscess in patients with primary spondylodiscitis (*p* < 0.05). Vertebral bone destruction was more severe in group NS (31% less than 75% of bone left) than in group S (12% less than 75% of bone left; *p* = 0.020). There was no difference in disc destruction between the two groups (*p* > 0.523) (Fig. 2). The occurrence of an epidural abscess correlated significantly with the occurrence of a paraspinal abscess: *r* = +0.260, *p* = 0.002, but did not correlate with vertebral bone destruction (*p* > 0.05). A significant correlation could be shown between a paraspinal abscess and vertebral bone destruction (*r* = +0.168, *p* = 0.046). Vertebral bone destruction tends to be more severe in patients with Staphylococcus aureus infection: in 25 vs. 33% of patients less than 75% of bone was left (*p* > 0.05).

The duration of hospital stay was longer in the patients who underwent prior surgery (15.8 ± 10 vs. 10.5 ± 9 days; *p* = 0.000). Survival rate in group S (98.2%) was higher than in group NS (87.5%, *p* = 0.024; Fig. 1). Patients with an epidural abscess tended to die more frequently than patients without: 10 vs. 5% (*p* > 0.05). Vertebral bone destruction correlated significantly with the rate of death: *r* = +0.047, *p* = 0.003. Thereby, the duration from the first diagnosis to death was 50.8 ± 72 days in surgically treated patients and 24.8 ± 21 days in conservatively treated patients (*p* > 0.05). For more details, see Fig. 1. Sepsis was detected more often in group NS (30%) than in group S (15%), although this was not statistically significant (*p* = 0.056). S. aureus presented the most common organism in intraoperative smear test and blood culture (Table 3), without significant microbiological differences between the groups (*p* > 0.05).

**Table 3:** Pathogen spectrum in intraoperative smear test and blood culture in all patients with primary and secondary acquired spondylodiscitis

		Group S	Group NS
Intraoperative smear test of surgically treated patients, *n* (%)	Positive	17/47 (36)	28/69 (41)
	*Staphylococcus aureus*	6/47 (13)	17/69 (25)
	*Staphylococcus epidermidis*	6/47 (13)	3/69 (4)
	*Escherichia coli*	1/47 (2)	2/69 (3)
	Others (e.g., *Propionibacterium* acnes, *Streptococcus gallolyticus*)	4/47 (9)	6/69 (9)
Blood culture of all patients, *n* (%)	Positive	13/55 (23)	45/104 (43)
	*Staphylococcus aureus*	4/55 (7)	19/104 (18)
	*Staphylococcus hominis*	1/55 (2)	2/104 (2)
	*Escherichia coli*	2/55 (4)	1/104 (1)
	Others (e.g., *Propionibacterium* acnes, *Streptococcus gallolyticus*)	6/55 (8)	23/104 (22)

*Staphylococcus aureus* presented the most common bacteria

*n* number of patients, *S* secondary acquired spondylodiscitis, *NS* primary acquired spondylodiscitis

**Fig. 3:**
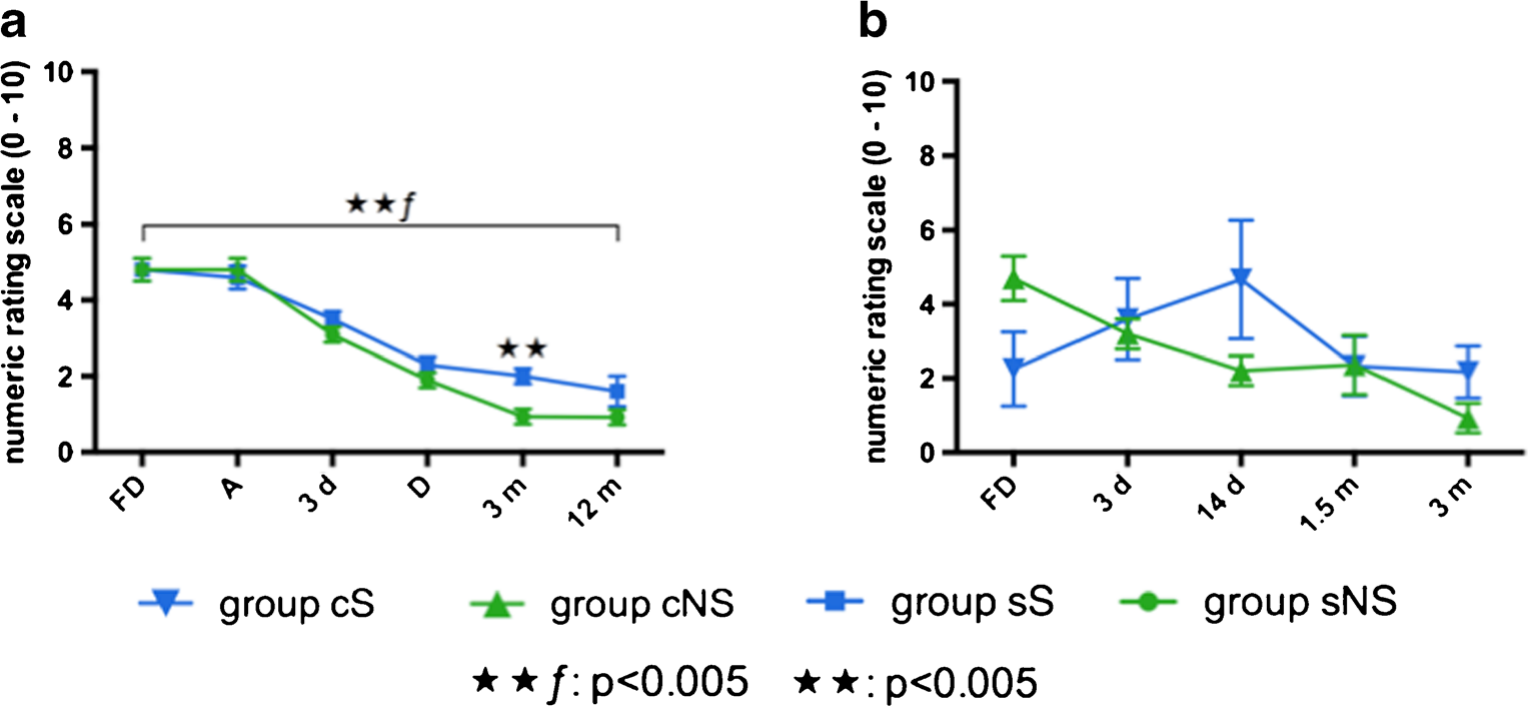
Overall pain on numeric rating scale (NRS) in a surgically (sS, sNS) and b conservatively treated patients (cS, cNS). *S* secondary acquired spondylodiscitis, *NS* primary acquired spondylodiscitis, *c* conservatively treated, *s* surgically treated. ★★ƒ: differences in follow-up: *p* < 0.005, ★★: differences between groups: *p* < 0.005. **a** *FD* first diagnosis, *A* day of admission, *3d* third postoperative day, D discharge, *3m* and *12m* *6m* and 12 months follow-up. **b** *FD* first diagnosis, *d* days after the first diagnosis, *m* months after the first diagnosis

**Fig. 4:**
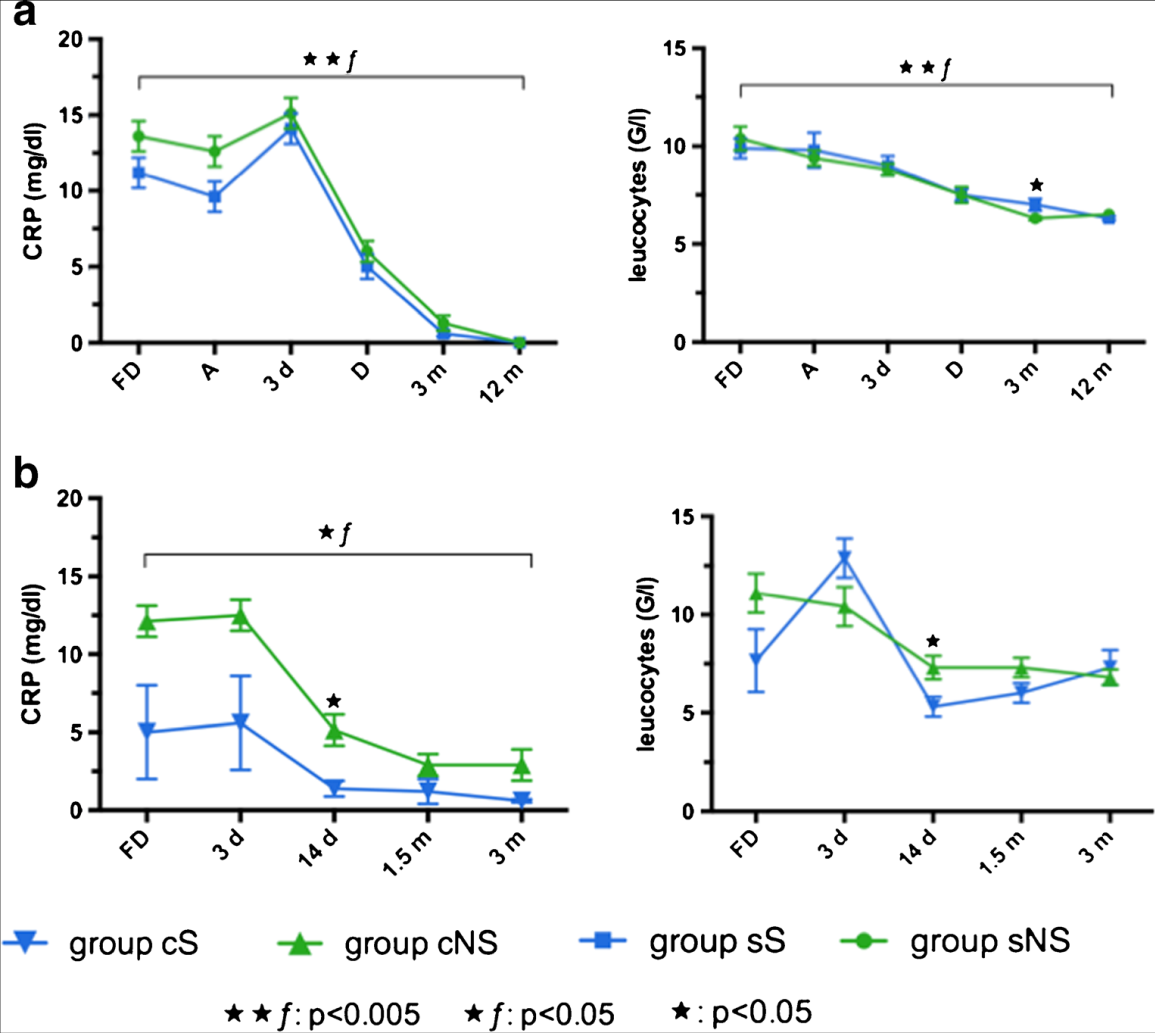
Inflammatory blood value in a surgically (sS, sNS) and b conservatively treated patients (cS, cNS). *CRP* C-reactive protein, S secondary acquired spondylodiscitis, *NS* primary acquired spondylodiscitis, *c* conservatively treated, *s* surgically treated. ★★ƒ: differences in follow-up: *p* < 0.005, ★ƒ: differences in follow-up: *p* < 0.05, ★: differences between groups: *p *< 0.05. **a** *FD* first diagnosis, *A* day of admission, *3d* third postoperative day, *D* discharge, *3m* and *12m* 6 and 12 months follow-up. **b** *FD* first diagnosis, *d* days after the first diagnosis, *m* months after the first diagnosis

In surgically treated patients (sS, sNS) significant differences were found in the extent of the operative procedure as described in Table 1 (*p* < 0.005), especially corpectomies were more often performed in group sNS than in group sS (*p* = 0.017). Duration of the first diagnosis until surgery was 15.8 ± 10 days in group sS and 15.8 ± 25 days in group sNS (*p* > 0.05). 64% in group sS and 67% in group sNS received primary conservative treatment before surgery (*p* > 0.05). Overall pain on NRS showed significant improvement after 12 months in both groups (*p* < 0.005), whereas NRS was rated significantly higher in group sS than in group sNS 3 months after surgery (p < 0.005) (Fig. 3). Laboratory parameters in both groups (sS, sNS) decreased significantly after surgery (*p* < 0.005) (Fig. 4). In conservatively treated patients (cS, cNS), differences in infection parameters could be shown after 14 days of the first diagnosis: CRP: cNS 5.1 ± 6 mg/dL vs. cS 1.4 ± 1, *p* = 0.08; leukocytes: cNS 7.3 U/L vs. cS 5.3±1 U/L, *p* = 0.044 (Fig. 4). No significant differences revealed between groups in NRS at the first diagnosis or in the follow-up visits of 3 months (*p* > 0.05, Fig. 3).

## Discussion

The authors present the results of the first retrospective study investigating the differences between primary and postoperatively acquired spondylodiscitis. Survival rate was significantly higher in patients with postoperative spondylodiscitis. MRI remains the most sensitive and specific imaging modality to detect spondylodiscitis and the infection of the adjacent tissue [4]. Epidural and paraspinal abscess and vertebral bone destruction was more severe in primary spondylodiscitis. Surgical treatment accompanied by broad-spectrum antibiotics resulted in significant reduction of overall pain and inflammatory blood values 12 months after surgery. No significant pain decrease could be detected in conservatively treated patients 3 months after the first diagnosis.

Infective spondylodiscitis can result in disc destruction, pathological fractures and abscess formations. A spinal epidural abscess is an infection involving the epidural space and has a high mortality rate [5]. Once a rare diagnosis carrying a poor prognosis, its incidence is rising dramatically. The development of an epidural abscess may be associated with various risk factors, such as advanced age over 65 years, immunocompromized state, smoking or diabetes mellitus. Furthermore, intravenous drug abuse has been associated with a high prevalence of epidural abscess commonly due to hematogeneous bacterial spread [6–9]. Drug abuse was more common in the patients who developed a primary spondylodiscitis. This could be a fundamental reason for the higher occurrence of epidural abscess in these patients and particularly for the higher mortality rate that is closely associated to infections with abscess formation [3, 10].

An epidural abscess may be associated with significant bone destruction resulting in instability and deformity of the spine [11]. In our study group, a significant correlation could not be shown, whereas a paraspinal abscess may induce vertebral bone loss. Instability establishes a major risk factor for developing a potentially neurological impairment. Then early surgical stabilization is insistently recommended to preserve neurological function [12]. Furthermore, vertebral bone destruction was more severe in primary spondylodiscitis, whereas differences in disc destruction did not occur between the two groups [11].

The most common causative organism in spondylodiscitis is Staphylococcus aureus which is responsible for widespread bone loss and bone destruction [13, 14]. Additionally, an increased long-term mortality rate, mainly due to sepsis, is reported in adult patients suffering from Staphylococcus aureus spondylodiscitis [14]. Thus, early surgical debridement is obtained to reduce the bacterial load and improve the antibiotic efficacy, especially in multiresistant Staphylococcus aureus infection [15]. Further causative organisms have been described and increased over the last few years, especially gram-negative bacteria. An important cause is the use of routine use of prophylactic antibiotics after spinal surgery [16]. Nevertheless, the pathogen that is responsible for spondylodiscitis can only be identified in approximately 35 to 50% of cases [17, 18]. In our retrospective series the pathogen could only be detected in 26% of blood culture and 39% of intraoperative smear test samples. This may be due to the fact that broad spectrum antibiotics have been applied in our outpatient clinics before a spondylodiscitis could be diagnosed accurately.

Surgery is indicated for neurological impairment, deformity, instability, medical intractable pain, and disease progression [19, 20]. The surgical goals are the debridement of infection, identification of pathogens, decompression of neuronal structures, and stabilization of deformed and instable segments. Depending on the location and extent of infection, various treatment options for spondylodiscitis are available. There is still controversy regarding the most adequate surgical approach [20–24]. Nevertheless, fusion is recommended in discitis with involvement of endplates of the vertebral body, whereas in intraspinal empyema dorsal decompression and evacuation alone may be sufficient [20]. Significant differences occurred in the extent of operative procedures in our study group. A higher spread of infection resulted in a more aggressive surgery. Cervical and thoracal corpectomy was performed more frequently in patients with primary spondylodiscitis. A minor part of patients were treated with debridement only because spondylodiscitis with liquefaction of endplates was present in over 90% of patients. Furthermore, patients who already had surgery were presumably screened earlier for spondylodiscitis as their counterparts. This may due to the fact that these patients are followed up routinely after their surgical treatment and therefore infection may be detected earlier in the infectious cascade.

In conclusion spondylodiscitis is a life-threatening and serious disease and requires long-term treatment. Our retrospective analysis demonstrates a significantly higher mortality rate in patients with primary spondylodiscitis. Primary spondylodiscitis is frequently associated with epidural and paraspinal abscesses and vertebral bone destruction. Thus, it seems that primary spondylodiscitis shows a more severe course than spondylodiscitis following spine surgery. Nevertheless, with current standards, prospective clinical trials will be mandatory to better understand the pathogenesis of spondylodiscitis and furthermore develop evidence-based treatment recommendations for these patients.
